# Continuous Droplet-Actuating Platforms via an Electric Field Gradient: Electrowetting and Liquid Dielectrophoresis

**DOI:** 10.1021/acs.langmuir.1c00329

**Published:** 2021-05-20

**Authors:** Iman Frozanpoor, Michael. D. Cooke, Vibin Ambukan, Andrew. J. Gallant, Claudio Balocco

**Affiliations:** Department of Engineering, Durham University, South Rd, Durham DH1 3LE, U.K.

## Abstract

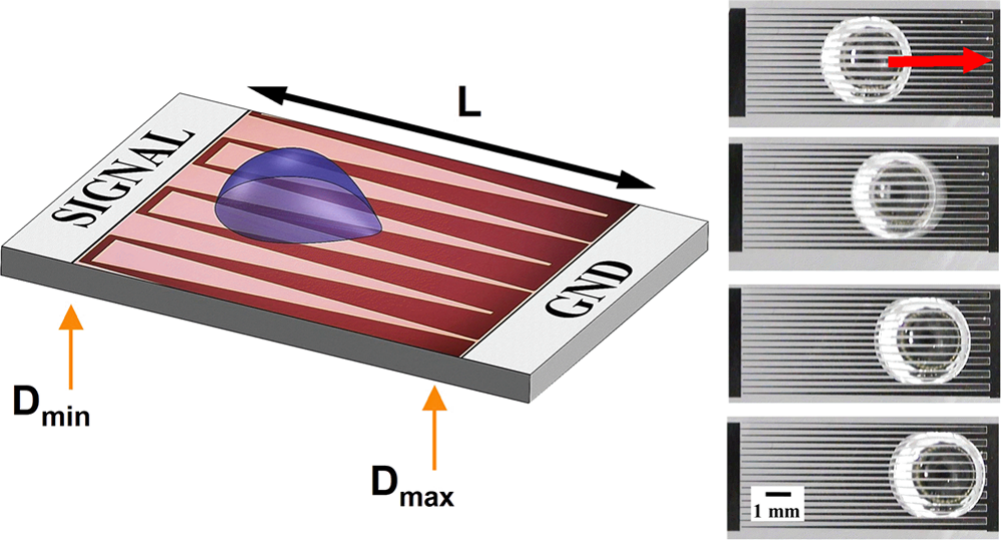

This work develops a technology for actuating droplets of any size without the requirement for high voltages or active control systems, which are typically found in competitive systems. The droplet actuation relies on two microelectrodes separated by a variable gap distance to generate an electrostatic gradient. The physical mechanism for the droplet motion is a combination of liquid dielectrophoresis and electrowetting. Investigating the system behavior as a function of the driving frequency identified the relative contribution of these two mechanisms and the optimum operating conditions. A fixed signal frequency of 0.5 kHz actuated various liquids and contaminants. Droplet actuation was demonstrated on several platforms, including linear, radial-symmetric, and bilateral-symmetric droplet motion. The electrode designs are scalable and can be fabricated on a flexible and optically transparent substrate: these key advancements will enable consumer applications that were previously inaccessible. A self-cleaning platform was also tested under laboratory conditions and on the road. This technology has significant potential in microfluidics and self-cleaning platforms, for example, in the automotive sector to clean body parts, camera covers, and sensors.

## Introduction

Since the pioneering work on microfluidics in the early 1990s, there has been an ever-increasing research focus on droplet manipulation in both open and closed configurations.^1−5^ However, improving and introducing new paradigms to minimize the device complexity is necessary to exploit this technology for large-volume applications.

Surface tension and capillary forces are the dominant factors in microfluidic systems due to the reduced operating scale. Electrowetting-on-dielectric (EWOD) and dielectrowetting (DW) are the two commonly used techniques to actuate droplets by electrical means.^6,7^ EWOD is a method to actuate conductive liquids by manipulating the interfacial surface energy in the presence of an electric double layer. A typical EWOD arrangement comprises of a conductive droplet sandwiched between two plates, where the top plate is a common ground and the bottom plate consists of an array of individual signal electrode pads.^8^ Nevertheless, other electrode configurations are also possible.^9^

Although EWOD has been widely studied,^10,11^ the method is restricted by limitations such as contact angle saturation and actuation incompatibility with non-conductive liquids.^6,7,12^ In contrast, DW has been gaining attention for overcoming the limitations of electrowetting.^13,14^ The dominant mechanism for DW is liquid dielectrophoresis (L-DEP), which exploits the electric bulk force produced near the liquid–solid interface of a droplet by applying a non-uniform electric field.^15^ Droplet manipulation with L-DEP has attracted a great deal of research interest, notably in the fields of optofluidics and lab-on-a-chip microfluidics.^16−19^

Recently, L-DEP actuation on a single plate using interdigitated electrodes (IDEs) design was demonstrated for splitting and transporting a variety of liquid droplets.^20^ This study was followed by investigating antibiofouling performance with a slippery lubricant-infused surface.^21^ Later, a programmable droplet-actuating platform based on L-DEP demonstrated the droplet actuation with varying volumes using an iterative fractal approach.^22^ Furthermore, the exploitation of high electric fields using IDEs with a small gap distance reduced the operating voltages (100 V or less).

The actuation of sessile droplets using EWOD and DW can be explained through the asymmetric electrostatic forces changing the contact angle on one side of the droplet, thus causing motion. An array of electrodes can be situated on a single plate and controlled using an electronic control system.^22^ The application of this technique on a large surface for a cleaning platform is complicated and costly and may also require a droplet sensing method within a feedback control loop. A few examples are vision systems, fluorescence spectroscopy, capacitive sensing, and impedance measurements.^23−26^ This limitation is primarily driven by the IDEs fixed surface area and varying droplet volume.

Continuous electrowetting is recommended in large-scale platforms for its simplicity and scalability. The initial attempts showed that continuous actuation was feasible using liquid metals in a closed channel.^27^ The electrowetting technique relied on the voltage drop across a thin layer of aqueous electrolyte to produce droplet motion. The introduction of nonlinear circuit elements enabled the continuous droplet actuation. For example, embedded diodes achieved continuous droplet motion using induced electro-osmotic and electrowetting effects.^28−31^

Furthermore, the continuous droplet actuation has been reported without requiring external input energy using a wettability gradient.^32^ The bidirectional droplet motion based on the gradient liquid-infused surface was demonstrated for long-distance droplet actuation.^33^ There are, likewise, new methods to transport microscopic liquid layers based on a unique topological structure.^34^ The topological fluid diode enabled long-distance directional liquid transport.^34^

Here, we demonstrate a continuous droplet motion based on variable interdigitated electrodes (VIDEs). The VIDEs approach represents a significant simplification compared to the traditional electric methods, leading to advantages in terms of reduced costs, control system requirements, and reliability on different scales. The foremost advantage is actuating droplets with different volumes without a control system. Additionally, the VIDEs can transport dielectric liquids, an operational limitation of the embedded diodes that require conductive liquids. The technological advancements presented here introduce a continuous droplet motion for various applications, including a cleaning platform for optical sensors and cameras, in addition to other chemical and biological devices based on droplet-based microfluidics.

## Theoretical Background

The combined working mechanism for DW and EWOD can be explained by the Korteweg–Helmholtz equation of liquid body force density.^35^
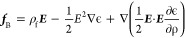
1Here, ρ and ρ_f_ are the density and free electric charge density of the liquid, respectively, ϵ is the liquid permittivity, and *E* is the electric field. The bold letters are vector quantities. EWOD and DW effects are frequency-dependent, and thus, the applied signal frequency determines the droplet actuation mechanism. Note that a greater EWOD effect is possible using a liquid with higher electrical conductivity. However, the ionic conductivity above a critical frequency is negligible, and the liquid behaves as a dielectric (ρ_f_ = 0). The electrostriction term is similarly ignored when the liquid is incompressible.^35^ Therefore, the indications from eq 1 is that the larger values of the electric field and liquid permittivity generate a greater L-DEP force.

**Figure 1:**
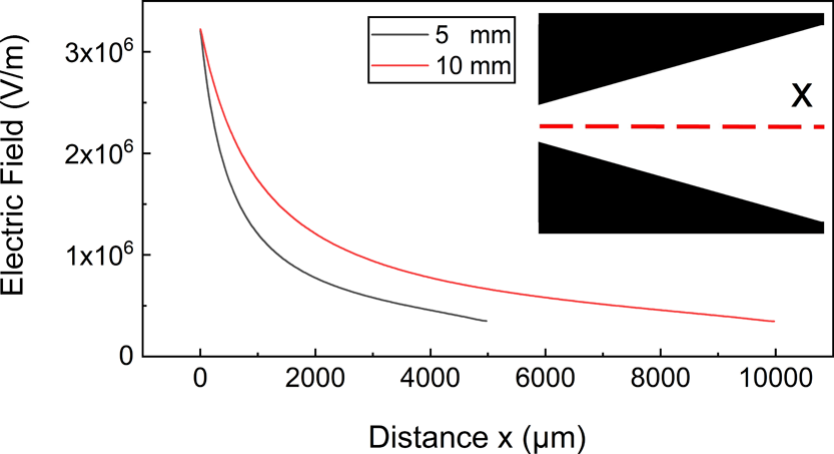
2D COMSOL Multiphysics simulation results showing the distribution of electric field between the electrode fingers at two different lengths (10 and 5 mm). The gap distance was between 200 and 20 μm. The applied voltage was 75 V, and the subfigure shows the cut plane “x” from where the readings were taken.

The VIDEs exploit the electric field’s favorable scaling by varying the electrode gap distance to generate an electrostatic net force, thus causing a continuous droplet motion. Figure 1 elaborates on the working mechanism using a two-dimensional (2D) COMSOL Multiphysics simulation to show the change in the electric field across the electrode. The electric field decays faster with shorter electrodes, resulting in a higher field gradient and net force. The dielectric breakdown was a design constraint and, therefore, very high voltages or a gap distance lower than 20 μm was avoided. Please refer to the Supporting Information for more details about the simulation setup and boundary conditions.

## Experimental Section

### Experimental Setup

The experiments were carried out in a cleanroom maintained at 20 °C. An alternating current (AC) signal was supplied from a function generator to a signal amplifier and then transmitted to the device. The experimental setup consisted of a testing station with pogo pins for electrical contacts. The testing station was on a leveled bench, and the signals were monitored using an oscilloscope. A microcontroller drove a simple relay module via three reed relays for switching the electrical signals. MATLAB was the interface to connect, control, and save the media files. The actuation time was determined from the videos to calculate the actuation speed. The droplet volume was regulated using a micropipette (±0.1 μL). Please refer to the Supporting Information (see Table S1) for the list of testing liquid and their properties.

### Design and Fabrication

**Figure 2:**
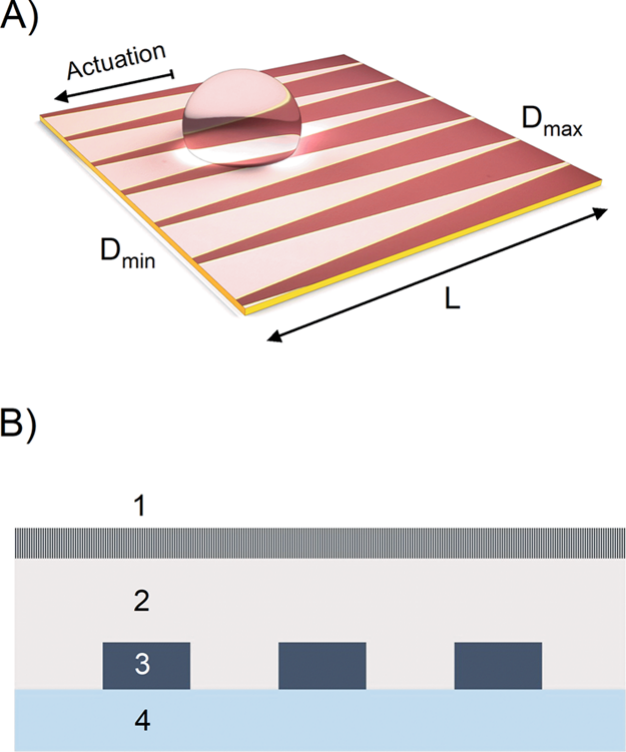
(A) General overview of the device moving a droplet. The important parameters are the electrode length (*L*) and a varying gap distance (*D*_n_). The electrodes are connected to a DC or AC voltage source to generate a variable non-uniform electric field along the length of the device. (B) 2D schematic of a typical device: (1) hydrophobic layer, (2) insulating layer, (3) electrode patterns, and (4) substrate.

The typical VIDEs consist of interdigitated electrodes with a variable gap distance (*D*_n_) and length (*L*), as depicted in Figure 2A. The device incorporates four separate layers (see Figure 2B). The first layer was a substrate (borosilicate glass), the second layer was an array of VIDEs (aluminum, 70 nm in thickness), and an insulating layer protected the electrodes. Photosensitive epoxy resin (SU-8) with a nominal thickness of 0.5 μm was selected here. Lastly, the SU-8 layer was functionalized with a hydrophobic self-assembled monolayer (SAM), octadecyltrichlorosilane (OTS), to obtain a hydrophobic top-layer for better performance with a contact angle of 110° (±4°). The OTS coating is widely used in electrowetting,^36,37^ and several other studies have already explored fabricating different SAM-functionalized SU-8 layers.^38−40^ Please refer to the Supporting Information for more details on the fabrication process.

The surface modification using a lubricant layer reduced the contact angle hysteresis associated with the pinning forces at the droplet contact line.^41^ Oil-based lubricant layers are commonly used to produce reversible spreading of the droplets in low-voltage electrowetting studies.^42−44^ We considered this approach to take accurate measurements using lower voltages. The selected lubricant layer was mineral oil, with an estimated thickness of 100 μm. The thickness of the oil layer was controlled by regulating the oil-injected volume over a confined area and then spin-coated to aid uniformity. The surface treatment modified the droplet-sliding angle (with a volume of 15 μL) from 15° to 1°. A superhydrophobic coating using SAM OTS is an alternative method to minimize the contact angle hysteresis without using any lubricant treatment.^38^ Furthermore, the actuation performance is dependent on the applied voltage, in which higher voltages can be employed for less hydrophobic surfaces up to the dielectric breakdown limit.

### Signal Management

**Figure 3:**
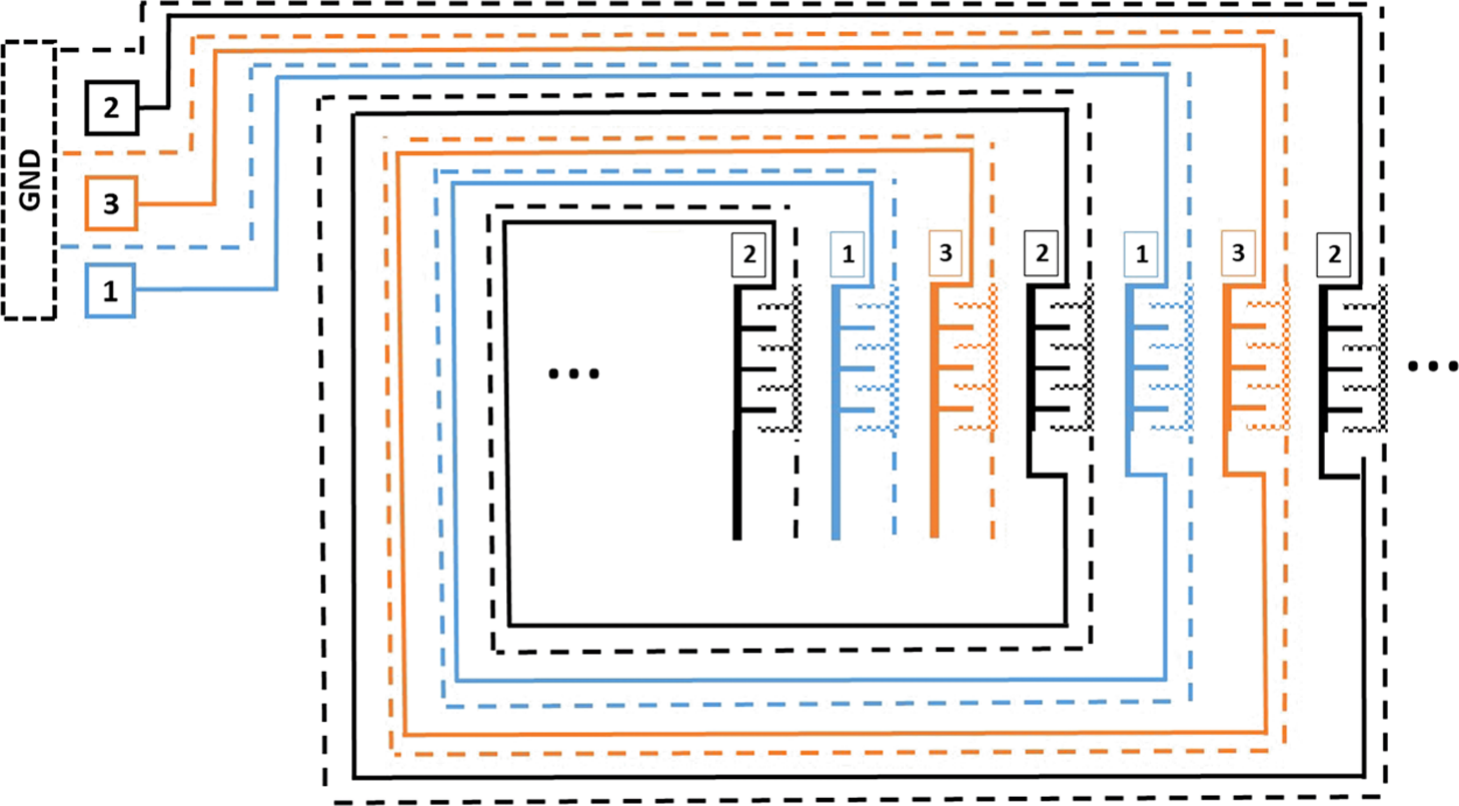
Interlinked signal flow between three sets of VIDEs. The multiplexing technique is similar to a multiple path maze that follows a spiral loop, with each pattern consisting of a common ground terminal. This approach requires only a single-layer photolithography process to connect multiple electrodes, thereby reducing the costs and fabrication complications of a multilayer design.

Droplet actuation on a large scale often requires a multilayer structure for electrode contacts, with signal management complications. The embedded signal patterns connect three separate paths (signal and common ground) from a source to any number of electrodes, removing the design requirement to fabricate many electrical contact points (see Figure 3). Combining the multiplexing technique shown here with the electrode design shown in Figure 2 can produce droplet actuation without size limitations.

## Results and Discussion

### Spontaneous Droplet Actuation

**Figure 4:**
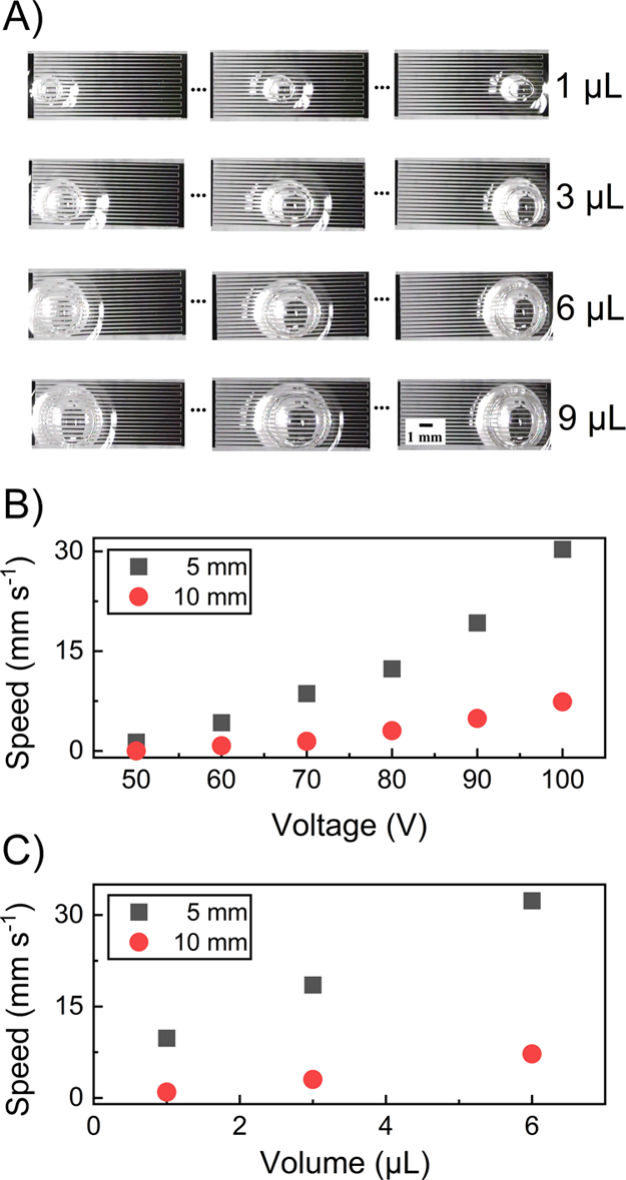
Experimental results characterizing the average droplet speed along the length of an electrode pad. (A) Top view images of droplets with different volumes moving on a typical VIDEs (*L* = 10 mm and *D*_n_ = 20–200 μm). (B) Comparing the actuation speed at different voltages with a fixed frequency of 20 kHz. The testing liquid was DI water with a volume of 6 μL, verified on two different pad size lengths. (C) Analysis of the actuation speed for different volumes of droplets with a fixed voltage of 100 V at a signal frequency of 20 kHz.

The ability to manipulate droplets of any size is a fundamental requirement for droplet-based microfluidics. Compared to other droplet-actuating methods, the electric-based platforms are not well suited to meet this critical performance criterion.^45^ Here, the continuous droplet actuation is verified with different volumes (see Figure 4A). The variable gaps in the VIDEs produced a net force across the electrode pad to initiate the droplet motion regardless of its position or size. Furthermore, the electrode patterns strip the need for a complex control system or the necessity to fabricate a large array of small electrodes, thereby reducing the overall complexity and costs.

From an application perspective, lower operating voltages are always desirable to avoid complex electronics and to aid electromagnetic compatibility. The introduction of the lubricant layer reduced the surface adhesion, resulting in lower operating voltages (as low as 30 V). However, the actuation on a plain OTS surface was only possible at higher voltages (100 V or more). Additionally, applying a modulated pulse AC signal (2 Hz) resulted in a smoother actuation for higher voltages or a step-by-step motion across the VIDEs using lower voltages (see Movie S1). Two electrode geometries with different lengths were tested to investigate the effect of applied voltage (see Figure 4B) and droplet volume (see Figure 4C) on the device’s performance. The experimental results in Figure 4 are based on a lubricant surface treatment to minimize the applied voltage.

The droplet size has a major impact on the actuation speed. Droplets with different volumes were tested to investigate the influence of the droplet size on the actuation speed. The actuation process required the droplet to be over at least one pair of VIDEs; therefore, in the current design, the droplet diameter had to be no less than 500 μm. However, a larger net force is generated when a bigger droplet is situated over multiple VIDEs.

The experiments verified that the shorter electrode delivered a better performance. The enhanced performance was because of the sharper changes in the electrode gap distance, producing larger forces. In contrast, the shorter electrodes cover a smaller surface area that requires an electronic switching method for larger platforms.

### Frequency-Dependent Actuations

The frequency-dependent analysis of aqueous droplets is critical to better understand the relationship between EWOD and L-DEP.^46,47^ Electrowetting and L-DEP actuation mechanisms dominate microfluidics in low- and high-signal frequencies, respectively. The utilization of the VIDEs allows the integration of L-DEP and EWOD domains onto a single device using a suitable signal frequency. A dielectrophoretic response was generated using a variable electric field above the critical signal frequency. Alternatively, a variable electric double layer effect was obtained in a conductive liquid using a signal below the critical signal frequency.

**Figure 5:**
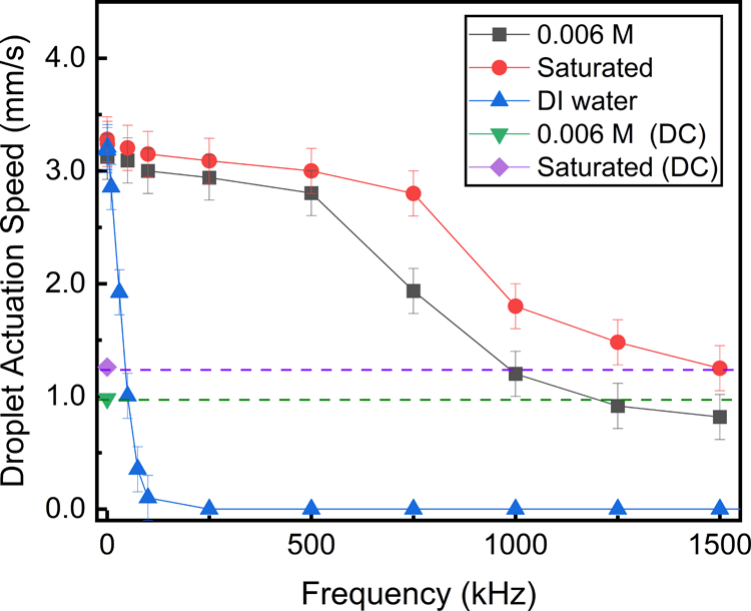
Frequency-dependent study for water and KCl solutions with different concentrations tested using DC and AC signal frequencies. The fixed electrode geometry has dimensions of *L* = 10 mm and *D*_n_ = 20–200 μm.

The water-based solutions with a different electrical conductivity were tested at room temperature using a wide range of signal frequencies (0.5 kHz to 1.5 MHz at 75 V RMS and a DC voltage applied at 75 V). The results are summarized in Figure 5. The testing liquids, saturated potassium chloride (KCl) solution and DI water, represent the two extreme examples of electrical conductivity, and 0.006 M KCl solution had similar properties to the natural rain.

The highest droplet actuation speed was in the low-frequency spectrum. For instance, the highest dependence on the frequency for DI water was registered between 0.5 kHz and 10 kHz. This is expected as the critical frequency for DI water is around 5 kHz.^47^ However, the critical frequency can slightly vary depending on the device parameters, such as the insulating thickness and the electrode gap distance. The KCl aqueous solutions behave differently because their conductivities are much higher than that of DI water, with the estimated critical frequencies being more than 500 kHz, as experimentally reported elsewhere.^47^

**Figure 6:**
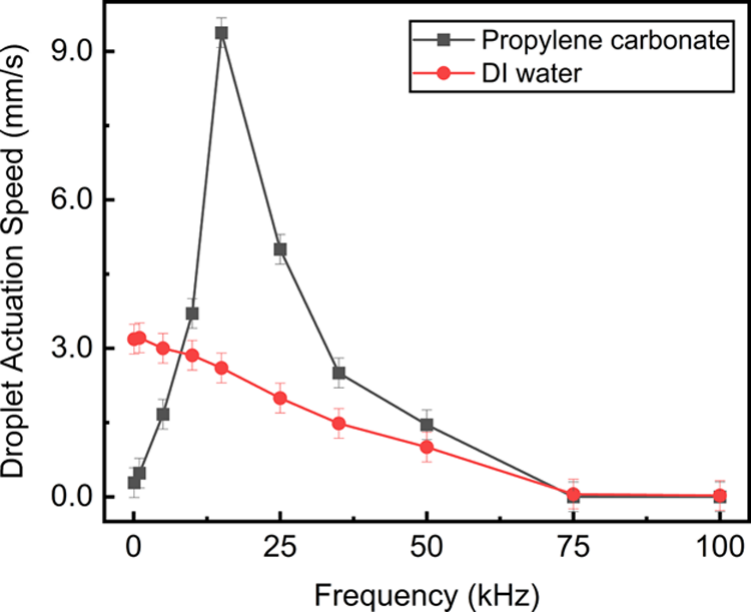
Testing results of dielectric liquids. The average droplet actuation speed is demonstrated here, highlighting the optimum applied signal frequency for each liquid. The applied voltage was 75 V.

The testing of dielectric liquids highlighted the optimum frequency at which the liquid experienced the highest dielectrophoretic response. The testing results for the dielectric liquids are shown in Figure 6. The actuation of dielectric droplets (i.e., propylene carbonate) was possible at lower voltages due to their superior chemical properties, such as high surface tension and large relative permittivity.^13,20^ The highest frequency response for the propylene carbonate was at 20 kHz, which was consistent with that of previous studies.^20^

The liquid’s electrical conductivity and permittivity change the critical frequency, meaning that the dielectrophoretic response is different for every liquid. Furthermore, employing a DC voltage resulted in virtually no actuation for dielectric liquids and lower performance for conductive liquids. Moreover, depending on the application, a DC voltage source might be favorable because of simpler control requirements.

### Large-Scale Droplet Actuation

Manipulating droplets using simpler and cheaper techniques is central to many lab-on-a-chip and surface-cleaning platforms. A large-scale device with the interlinked signal design (see Figure 3) allowed parallel and continuous droplet actuation with different volumes without increasing the complexity or fabrication costs.

**Figure 7:**
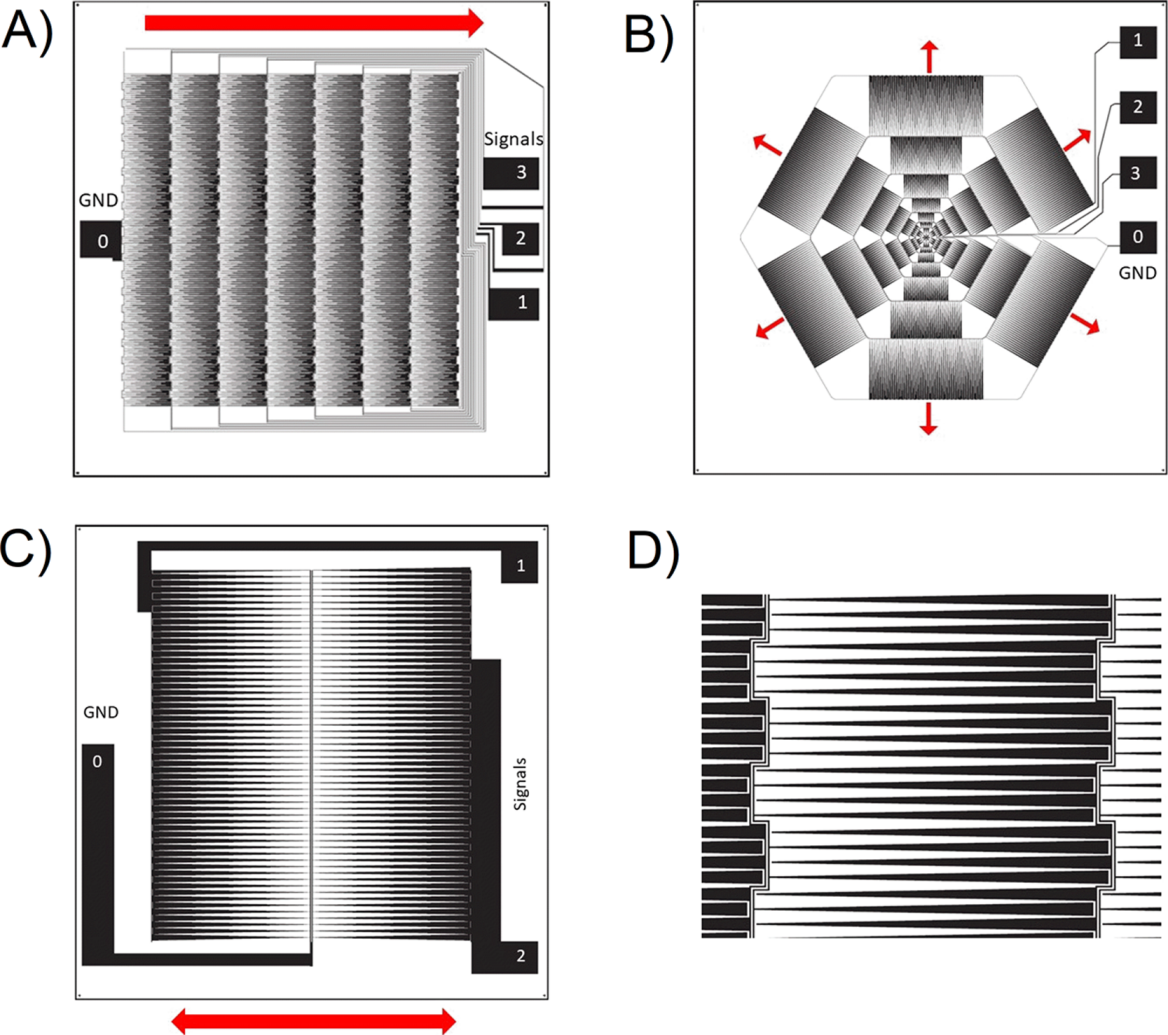
Three electrode designs to generate a continuous droplet motion on a large scale. The direction of the droplet motion is highlighted with arrows. (A) Linear droplet motion using seven VIDEs based on the interlinked flow signal shown in Figure 3. (B) Radial-symmetric droplet motion based on the interlinked flow signal. (C) Bilateral-symmetric droplet motion without an electronic control system. (D) Zoomed-in image, showing the overlapped region between two electrode pads.

A fixed sine-wave signal frequency of 0.5 kHz was selected for the experiments. The active area of the electrodes was approximately (4 × 4 cm). Two designs are suggested here (see Figure 7A,B), integrated with the interlinked signal design (shown in Figure 3), to demonstrate linear and radial-symmetric droplet motions on a large scale. There is also a small overlap region between every VIDEs for a smooth droplet actuation (see Figure 7D).

**Figure 8:**
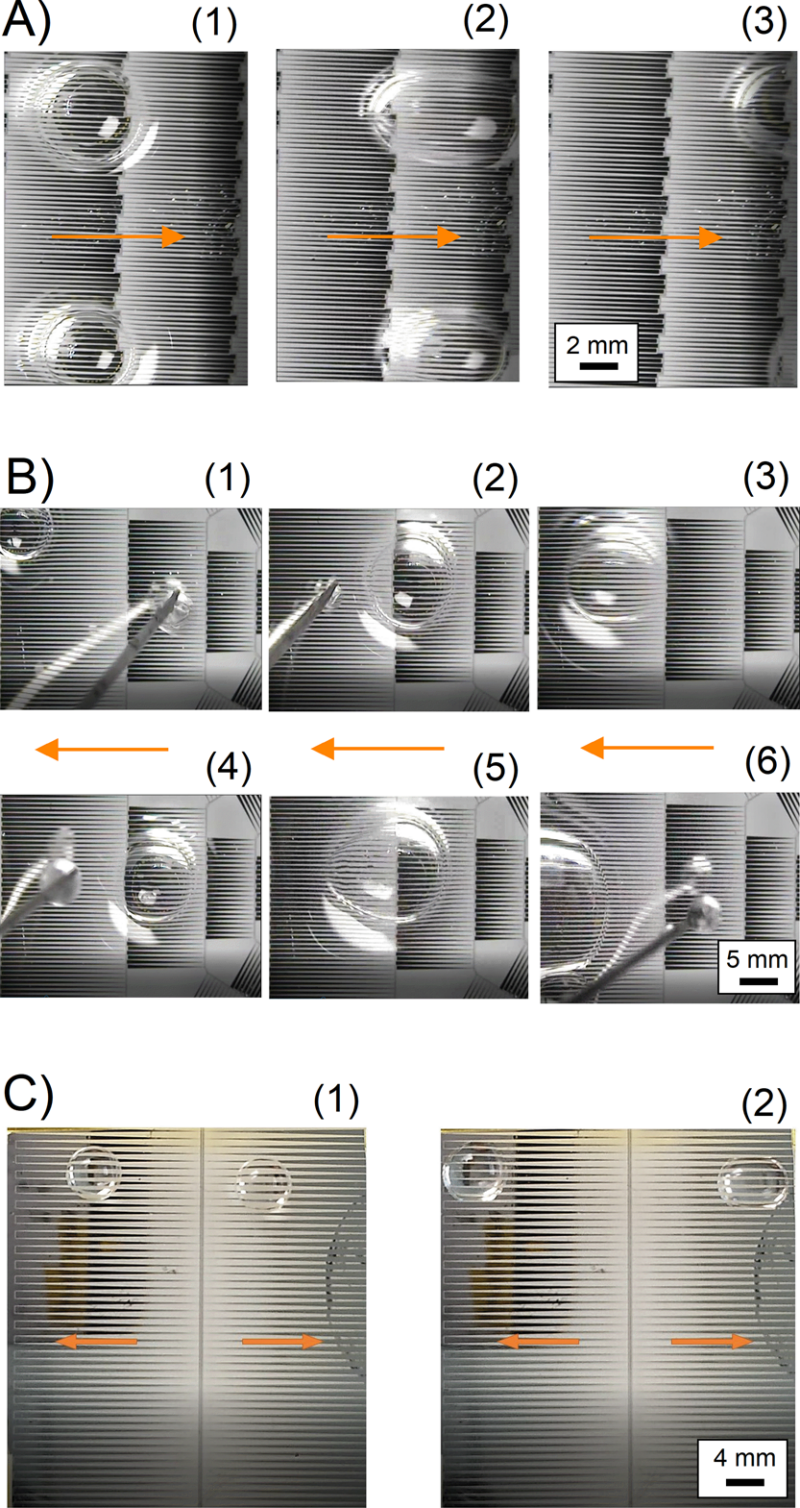
Summary of the test results showing DI water droplets moving on the surface with oil lubricant treatment. There is a 5 s delay between every switching step. (A) Linear droplet motion based on the design shown in Figure 7A using 75 V. (B) Radial-symmetric droplet motion based on the design shown in Figure 7B using 90 V. (C) Bilateral-symmetrical droplet motion based on the design shown in Figure 7C using 90 V.

The linear droplet motion (Figure 7A) uses an array of shorter VIDEs to actuate a range of droplets, resulting in a higher actuation speed. The design is suitable for a large-scale cleaning platform, where the linear droplet motion is appropriate, that is, for automotive applications (see the test results in Figure 8A). The radial-symmetric droplet motion (see Figure 7B) is carried out on a sunflower design with different electrode lengths. The droplet motion is validated by introducing random water droplets on the surface with different volumes so that the device moves them to the outer regions for disposal (see Figure 8B). This design is ideal for applications where radial-symmetric droplet motion is necessary, such as cleaning electronic sensors on a flat surface. The surface area of the blank gaps in the design increases in the outward direction by the golden ratio. The droplets in the inner regions are continuously transported to the outer areas to form larger droplets, eliminating the impact of large gaps in the outer areas of the device. Additionally, the droplet size must be smaller than the length of the smallest VIDEs. Otherwise, the droplet goes back and forth between the smaller pads. The alternative solution dedicates a separate voltage signal for every electrode pad.

A large-scale design is presented in another approach based on the bilateral-symmetric droplet motion without any electronic control systems (see Figure 7C). The simple design requires only two signals and a common ground to operate (see the test results in Figure 8C). The opposing electric forces in the center of the device (between the two VIDEs) could generate a lag in the actuation process and thus prevent any motion. An effective solution is a basic switch mechanism to eliminate the opposing forces (i.e., by switching the VIDEs separately ON-OFF, OFF-ON, ON-OFF ...).

Although previous studies demonstrated droplet motion in a discrete manner, the scale of the operation was limited, with applied voltages in excess of hundreds of volts. The fixed signal frequency was another simplifying factor to minimize the effect of electrical conductivity on the performance. Furthermore, the fabrication of a large-scale device using transparent electrodes expands the application of this technology, that is, to clean optical sensors or cameras. Please refer to the Supporting Information for examples of transparent devices (see Figure S1 and Figure S2).

### Surface Cleaning Application

The application of this technology on a large scale, that is, in the automotive industry, requires cleaning from contaminants such as dirt, soil, sand, and so on. The image clarity received by the sensors and cameras under a wide range of environmental conditions is critical for road safety.^48^

**Figure 9:**
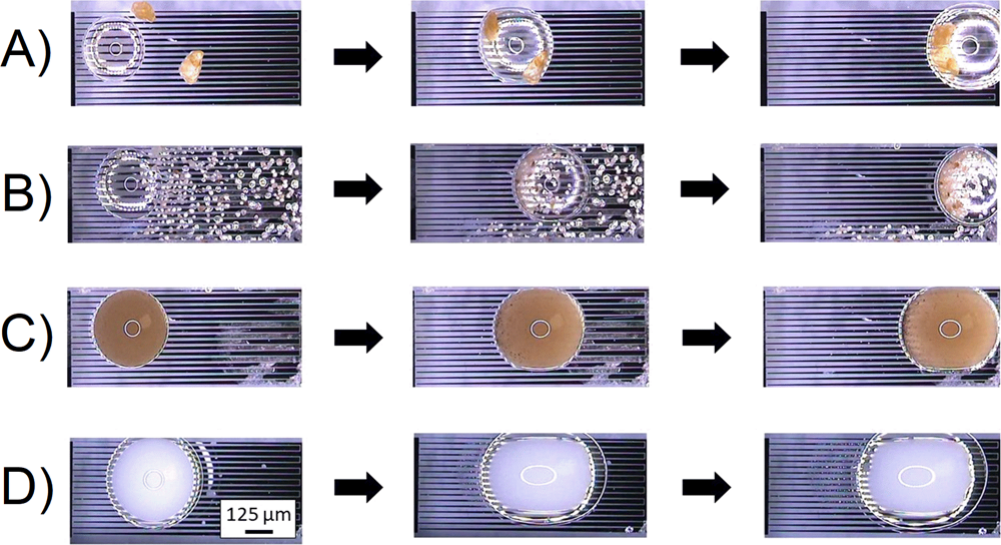
Summary of the test results, showing the top view of different droplets moving on the surface with lubricant treatment. (A) Rain droplet moving dirt contaminants (700–1000 μm), (B) rain droplet moving sand (10–150 μm), (C) actuation of suspension of a muddy water droplet, and (D) actuation of semi-skimmed milk droplet.

The signal frequency was fixed to 0.5 kHz at 100 V. We verified the removal of sand and dirt contaminants with a diameter of 10 μm to 1000 μm using a rainwater droplet (see Figure 9A,B). Additionally, the removal of a typical suspension liquid (mud rain) was demonstrated (see Figure 9C). The actuation of mud rain, sand, dirt, and rainwater showed the application of this technology for a practical scenario, for example, a car traveling on the highway. The actuation of droplets in microfluidics has extensive biological applications.^49^ The VIDEs actuated semi-skimmed milk droplets to demonstrate the flexibility of the platform (see Figure 9D). Semi-skimmed milk contains fat, proteins, and vitamins, including A, B3, B5, and D.

**Figure 10:**
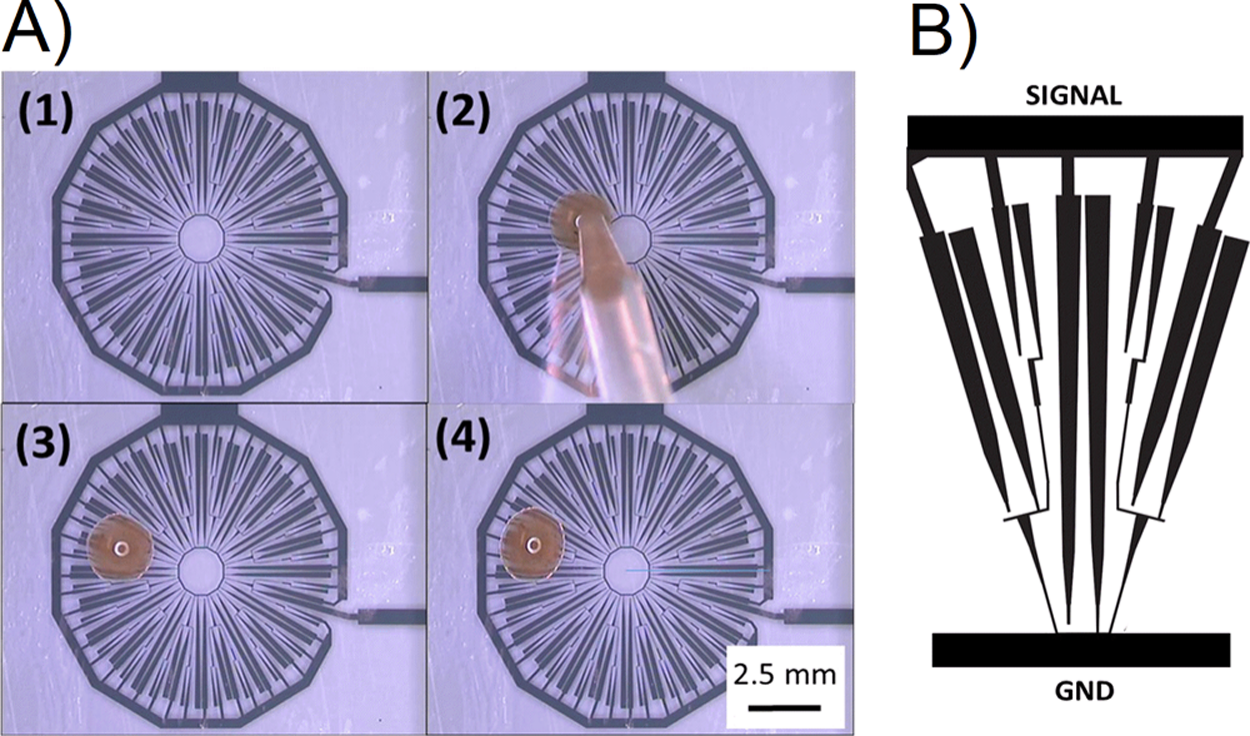
(A) Top view images (1–4) of a small-scale device (10 × 10 mm) that demonstrated a circular symmetrical droplet motion. The testing liquid was mud rain. (B) Part of the electrode design that consists of three VIDEs with different lengths.

A self-cleaning cover lens that systematically removes different contaminants and liquids without a control system is advantageous in many applications. An alternative approach to the previous designs is also proposed for a miniature cover lens (10 × 10 mm) using integrated VIDEs with different lengths. Figure 10 shows the testing results of moving a suspension of mud rain. The experiment verified the circular symmetric droplet actuation away from the center of the lens using a single voltage source.

The cover lens can be attached to the surface for easy integration with any device. The experiments verified the rapid cleaning of a camera lens positioned horizontally. Nevertheless, a simpler design similar to the one shown in Figure 9 is more suitable for an inclined surface where the actuation direction is fixed and linear.

A self-cleaning cover lens that prevents the build-up of contaminants could be an auxiliary add-on to sensors and cameras. Furthermore, the actuation of complex fluids (such as mud rain) is advantageous, which could either obscure the view or, when evaporated, leave a stain on the lens. Even though mechanical cleaning may still be necessary, minimizing its use for other solid contaminants is still a priority for many applications.

**Figure 11:**
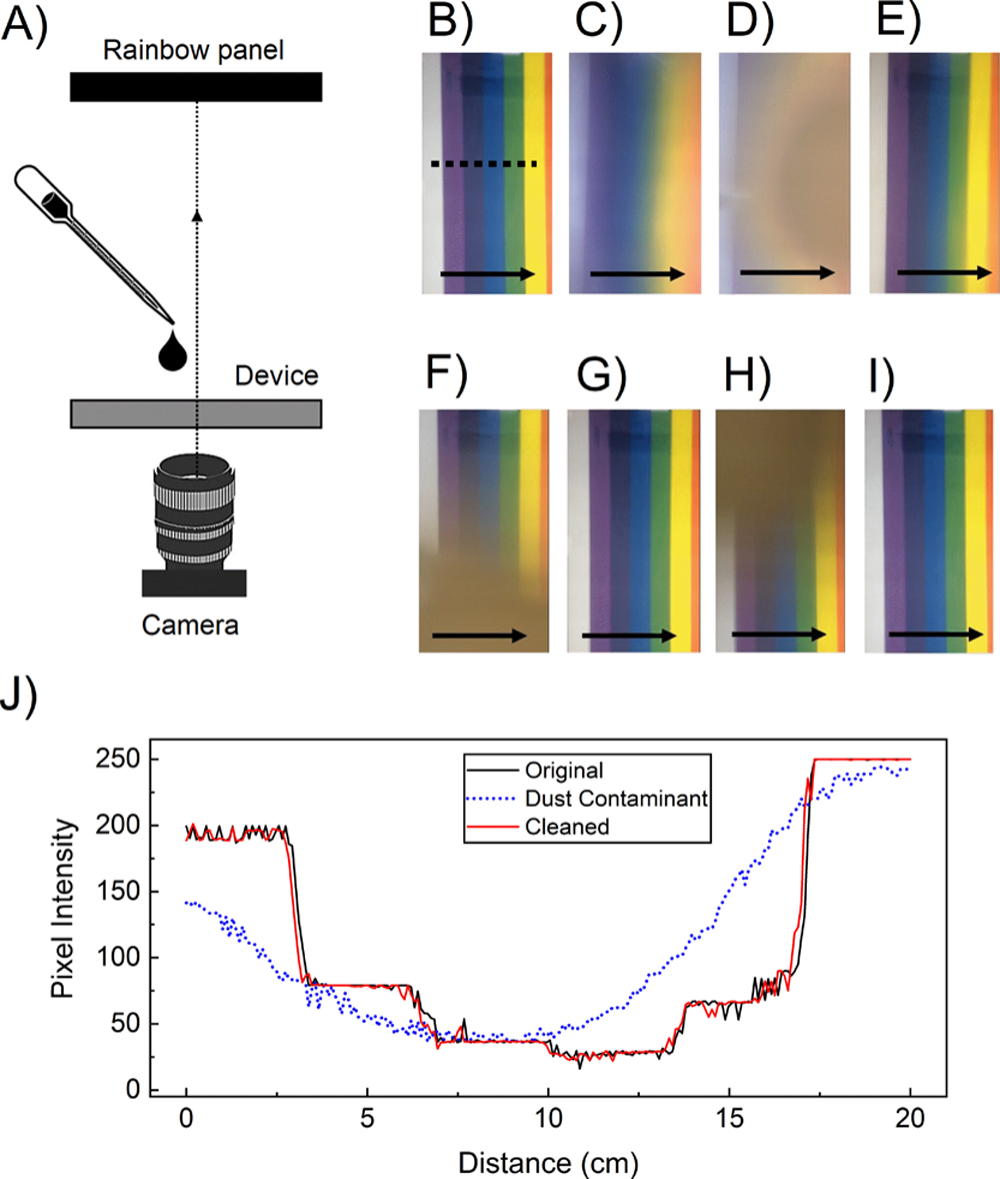
Summary of the test results for cleaning a camera cover lens. The black arrow shows the direction of the droplet actuation. (A) Overview of the experimental setup with a rainbow panel, device, and a camera, (B) clean surface, (C) adding dust particles to the surface, (D) adding a droplet, (E) cleaning the surface of the device, (F) adding another droplet to the surface, (G) cleaning the device, (H) adding a droplet, and (I) cleaning the device. (J) Demonstration of the cleaning process using a 2D plot showing the luminance across the rainbow pattern (from B–E).

The cleaning platform was mounted on a camera lens and tested (see the experimental setup in Figure 11A). The testing results verified the reliable and systematic cleaning of the surface against solid contaminants (see Figure 11B–E) and mud rain (see Figure 11F–I) to maintain a clear view during operation. The testing liquids were DI water and mud rain, yet other liquids are similarly compatible, including isopropyl alcohol. Figure 11J shows the luminance across the rainbow pattern during the cleaning process. The scanned regions are indicated with a dotted line.

There is a technological demand for an electronic self-cleaning platform to remove the surface contaminants on cameras, LIDAR, and sensors, which poses a growing engineering challenge to automotive manufacturers, specifically for self-driving cars.^48^ The cover lens was also tested on the road by mounting it on a camera and connecting it to a car battery via a power inverter. The device provided good visibility during the test when compared to a controlled camera without the VIDEs cover lens. The testing was carried out when the vehicle was stationary and similarly when on the road, moving at 40 mph. Please refer to the Supporting Information for more details (see Figure S3 and Movie S3).

## Conclusions

A novel method was successfully demonstrated based on the continuous actuation of droplets with different volumes (1–30 μL) using L-DEP and EWOD. The reported results verified the droplet actuation at lower voltages (as low as 30 V). At higher voltages (100 V or more), actuation speeds of up to 36 mm s^–1^ were registered for DI water (6 μL) on a 5 mm long electrode pad. A stronger electric field with a deeper penetration at a higher voltage may generate even larger forces, and therefore, further refinement is feasible.

The frequency-dependent study for different liquids at high- and low-frequency limits highlighted the best operating parameters. Furthermore, a fixed applied frequency (0.5 kHz) simplified the actuation process. This value was dependent on the liquid properties and may vary for other applications. Furthermore, the interlinked signal pattern was another simplifying addition for large-scale platforms.

The primary limitation of the VIDEs is the unidirectional droplet motion, limiting its application. However, bidirectional actuation is also feasible by using two sets of electrode patterns with an opposite variation of gap distance. Furthermore, a multilayer electrode design could also produce droplet motion in 2D.

The continuous droplet motion of VIDEs has several uses, notably in the fields of lab-on-a-chip microfluidics to transport droplets for analysis. The technology is similarly suitable for automotive applications in cleaning sensors and cameras. The droplet actuation on different scales promises significant advantages over the current technologies, including an overall reduction in the device complexity, operating voltage, and fabrication costs. The improvements presented here open many avenues for future innovative applications based on the VIDEs configuration.

## Supporting Information Available

Detailed fabrication process, testing liquids, details about the testing on the road, geometries of the VIDEs, and details about the simulation models (PDF)
Droplet actuation across the VIDE’s (MP4)
Device scalability (MP4)
Application of VIDEs in laboratory settings and on the road (MP4)
