# Supplementary material for: Continuous Droplet-Actuating Platforms via an Electric Field Gradient: Electrowetting and Liquid Dielectrophoresis
Source: Langmuir. 2021 May 20;37(21):6414–22. doi: 10.1021/acs.langmuir.1c00329 (PMC8397340; doi:10.1021/acs.langmuir.1c00329)
Supplement: Supplementary file 1 — la1c00329_si_001.pdf [file la1c00329_si_001.pdf]

Continuous Droplet Actuating Platforms via an Electric Field Gradient: Electrowetting and Liquid Dielectrophoresis

Iman Frozanpoor, Michael Cooke, Vibin Ambukan, Andrew Gallant, Claudio Balocco,*

Supporting Information

AUTHOR ADDRESS

Department of Engineering, Durham University, South Rd, Durham, DH1 3LE, UK.

Corresponding Author

Iman Frozanpoor - Department of Engineering, Durham University, South Rd, Durham, DH1 3LE, UK. **E-mail**: Iman.frozanpoor@durham.ac.uk

Table of Contents

The supporting information includes a detailed fabrication process, testing liquids, details about the testing on the road, geometries of the VIDE's, and details about the simulation models.

Table S1: Testing liquid and their properties.

Table S2: Electrode geometries and control system requirements.

Figure S1: Transparent device on the glass.

Figure S2: Transparent device on a flexible substrate.

Figure S3: Images showing the testing of a self-cleaning cover lens when on the road.

Movies S1: Droplet Actuation across the VIDE's.

Movies S2: Device Scalability.

Movies S3: Videos showing the application of VIDE's in laboratory settings and on the road.

Device Fabrication

The fabrication process was carried out in the engineering microfabrication laboratory at Durham University. Initially, borosilicate glass substrates were cleaned using a piranha solution (3:1 mix of sulfuric acid: hydrogen peroxide), then rinsed thoroughly using deionized (DI) water and dried with filtered nitrogen gas.

Standard photolithography was used to fabricate the electrodes. 70 nm of aluminum was deposited using E-beam evaporation. The samples were spin-coated with a positive photoresist (S-1813) and soft-baked at 95 °C for 3 min, then exposed to UV-light (80 mJ/cm^2). The samples were developed (MF-319) to get rid of the resin. In addition, the samples were baked at 115 °C for 3 min and then finally patterned using a wet etch process (aluminum etchant). Lastly, they were dipped inside a remover (1165), cleaned using water, and dried using nitrogen gas.

Transparent devices were also fabricated to demonstrate the range of applications for this technology. A particular interest is the utilization of the cleaning platform on a flexible substrate allowing the device to be mounted onto the curved surface. The fabricated transparent device (see Figure S2) was Indium tin oxide (ITO) on a PET flexible substrate.

Initially, the flexible substrates were cleaned in ultrasound using IPA solvents. The samples were spin-coated with a positive photoresist and soft-baked at 95 °C for 3 min, then exposed to UV-light (80 mJ/cm^2), followed by a developing process. The electrodes were patterned using a wet etch process using an ITO etchant. Lastly, the resist was removed, cleaned with water, and dried using nitrogen gas.

SU-8 2000.5 series was spin-coated (500 nm) on the electrodes followed by a soft-baked process at 95 °C for 1 min. The layer was then exposed with a dose of 60 mJ/cm^2, baked at 95 °C for 1 min, and developed for 1 min (EC solvent). Finally, the layer was rinsed with isopropyl alcohol

(IPA) and hard-baked at 155 °C for 20 min to ensure the layer is fully cross-linked. The typical thickness of the SU-8 layer was 500 nm, and a thickness of 1 μm was selected for devices tested at a higher voltage.

The solution of hexane/OTS (1 part of OTS in 2000 parts of hexane by volume) was prepared in a pre-cleaned glass beaker and sonicated for 10 min for better uniformity. The devices were then immersed in the OTS/hexane solution for 7 hours with no agitation. They were immediately dipped in hexane and dried with filtered nitrogen. The final step was to bake the samples on a hotplate for 15 min at 95 °C to complete the OTS polymerization process and eliminate any hexane residuals.

Experimental Method

The droplet speed was measured by determining the time and distance traveled over the VIDE's. The measured time was accurate down to 30 milliseconds, and the distance traveled along the electrode could be measured down to the microscales.

The testing liquids with their material properties are listed in Table. S1. The higher surface tension was important in both EWOD and L-DEP. The higher value of permittivity was crucial in the L-DEP, whereas the larger electrical conductivity was more dominant in the EWOD up to the saturation limit. The droplet actuation was also possible on a range of other liquids, including Isopropyl alcohol, typically found in cleaning fluids.

Table S1. Testing liquid and their properties.

Type of Testing Liquid	Electrical Conductivity	Permittivity	Surface Tension
Units	*μS/m*	*F/m*	*mN/m*
DI water	15	81.0	72.72
Rain water	10^6	80.0	72.72
KCL saturated	10^{10}	45.0	78.58
KCl 0.006 M	10^6	78.0	72.00
Propylene carbonate	NA	66.1	40.90
Semi-skimmed milk	10^5	60.0	38.00

Experimental Method (Test on the road)

The testing results are summarized in Figure S3. The device was powered by the standard car battery using an inverter, function generator, and a signal amplifier. Additionally, Raspberry Pi was streaming a live video into a web page. The camera lens was initially covered with mud and then systematically cleaned using rainwater. The testing was carried out on a plain OTS surface to mimic a real scenario. The downward droplet motion using a VIDE's (100 V at 0.5 kHz) was coupled with the gravitational forces to move the droplets rapidly. Furthermore, the device was also tested when the vehicle traveled at 40 mph on a rainy day, maintaining a clear view during the test. The wind shear force is substantial, preventing any droplets from sticking to the surface. Note that the applied voltage may vary depending on the testing parameters, including the vehicle speed and the precipitation rate. The summary of the test result is shown in Movie S3.

Electrode Designs

Table S2 summarizes the fabricated electrode geometries.

Table S2. The electrode geometries and control system requirements.

VIDE's Configuration	Variable Gap Distance, (μm)	Length (mm)	Control System Requirement
Linear VIDE's 1.0	D_{min} = 20<br>D_{max} = 200	5	NA
Linear VIDE's 2.0	D_{min} = 20<br>D_{max} = 200	10	NA
Linear VIDE's Multiple	D_{min} = 20<br>D_{max} = 200	5.5	3 control signals
Sunflower VIDE's	D_{min} = 20<br>D_{max} = 200	0.5, 1, 1.5, 2.5, 4, 6.5	3 control signals, but separate connections if there is a large variation in the droplet volume
Bilateral-Symmetrical VIDE's	D_{min} = 20<br>D_{max} = 600	20	2 control signals
circular Symmetrical VIDE's	D_{min} = 20<br>D_{max} = 150, 200	3, 2, 1	NA

COMSOL Multiphysics

The electrostatic interface on COMSOL Multiphysics 5.4 simulated the electric fields. COMSOL software essentially solves Poisson's equation. Further details and the governing partial differential equations (PDE's) are provided in COMSOL AC/DC module user's guide on the main website.[1] Because of the symmetry, only one set of VIDE's is considered here. The system parameters are two electrodes (signal and ground), with electrode gap distances from 20 μm to 200 μm at two lengths (10 mm and 5 mm). The potential on the grounded electrode is zero, and the effective voltage was set to 75 V on the signal electrode. COMSOL automatically selects the conservation of charge for the boundaries between the domains. Including the equations for charge conservation according to Gauss's law for the electric displacement field. Additionally, a zero

charge boundary is selected at the exterior boundaries. This boundary condition is similarly valid at symmetry boundaries where the voltage is symmetrical with respect to the boundary.

References

1. Electrostatics Theory. COMSOL Multiphysics, **2019**, https://www.comsol.nl/multiphysics/electrostatics-theory?parent=electromagnetics-072-162 (accessed 08/02/2021).

Supplementary Figures

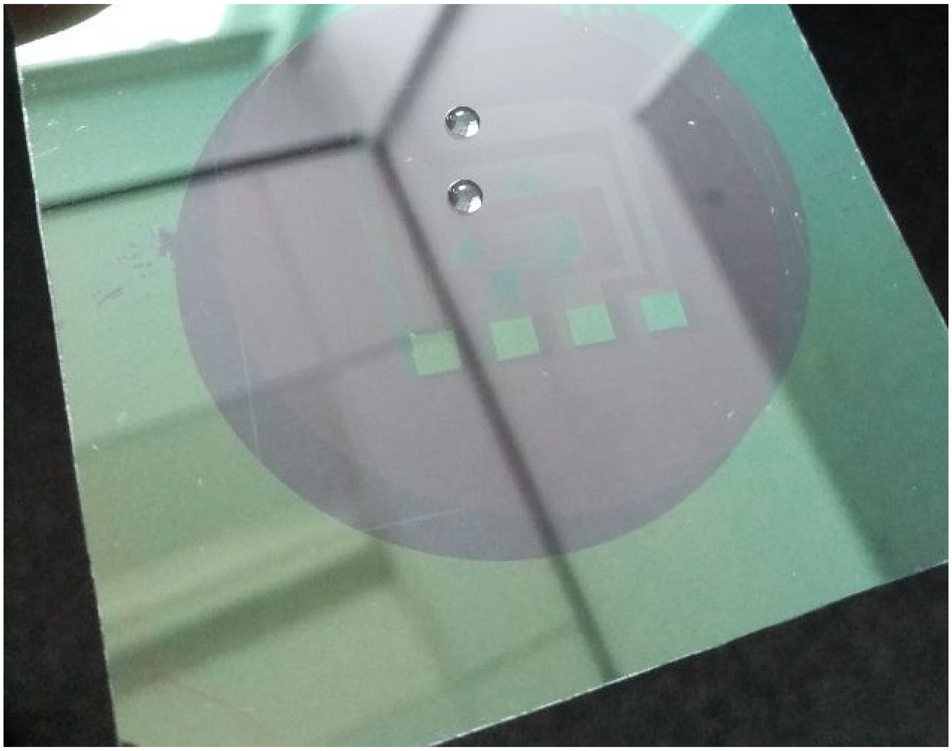

Figure S1. The transparent device on a glass substrate. ITO electrodes protected by SU-8 insulating layer and coated with SAM OTS.

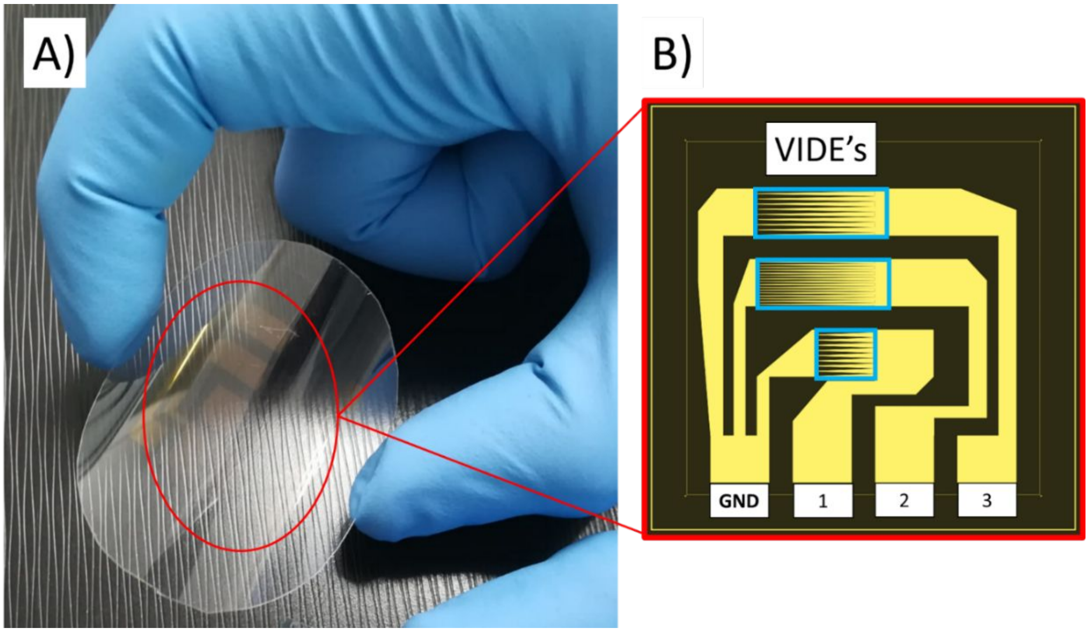

Figure S2. (A) Transparent flexible electrodes. The black mesh background and deflected light show the slightly shaded region with a layer of ITO electrodes. (B) The typical electrode mask design of VIDE's.

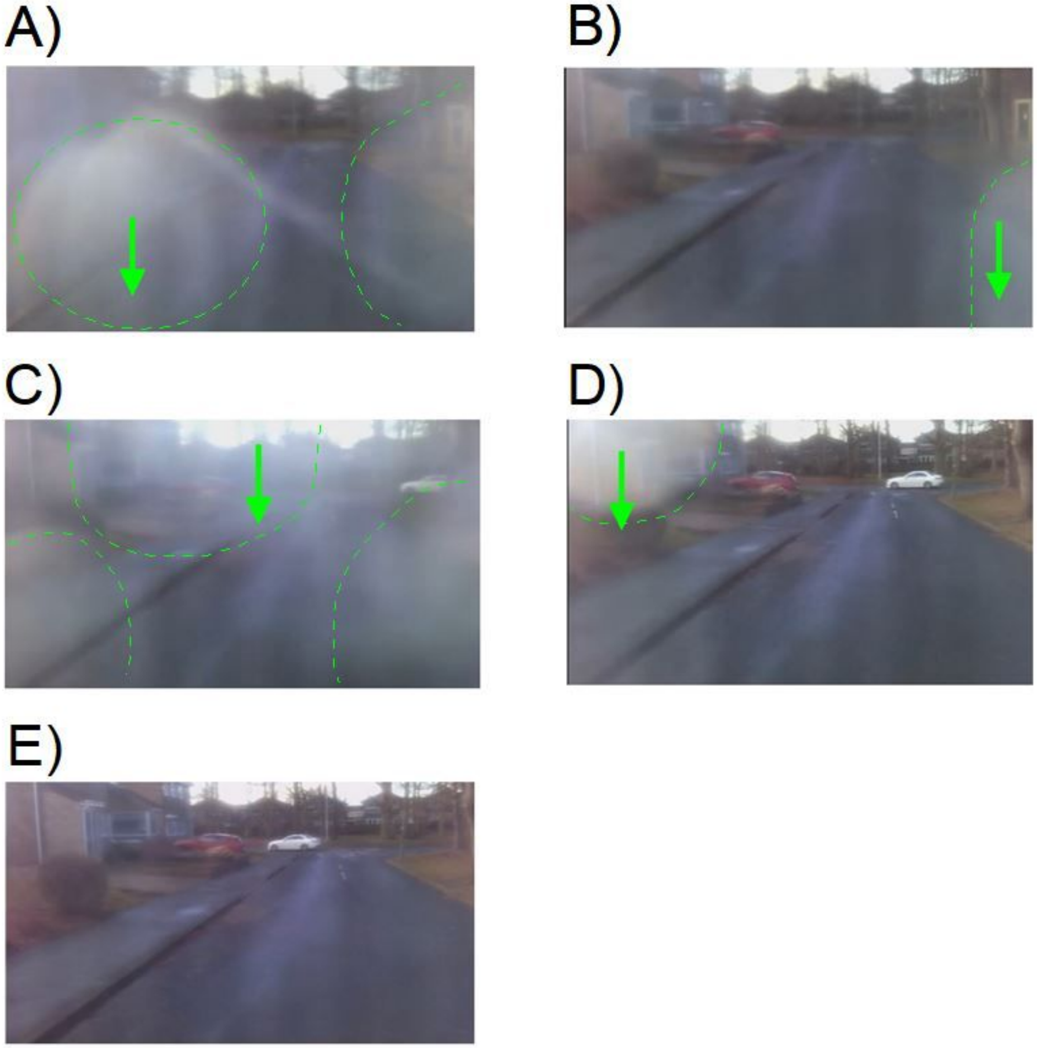

Figure S3. (A to E) shows the results for the testing on the road experiment, when the vehicle was moving at less than 10 mph. The downward droplet motion is highlighted in green. The droplet motion is assisted by the VIDE's, coupled with the gravitational forces.

Supporting Movies

Movies S1 Droplet Actuation

Movies S2 Device Scalability

Movies S3 Application of VIDE's

AUTHOR INFORMATION

Corresponding Author

Iman Frozanpoor - Department of Engineering, Durham University, South Rd, Durham, DH1 3LE, UK. **E-mail**: Iman.frozanpoor@durham.ac.uk

Present Addresses

Claudio Balocco - Department of Engineering, Durham University, South Rd, Durham, DH1 3LE, UK. **E-mail**: claudio.balocco@durham.ac.uk

Vibin Ambukan - Jaguar Land Rover Limited, National Automotive Innovation Centre, Coventry, CV4 7AL, UK. **E-mail**: vambuka1@jaguarlandrover.com

Michael. D. Cooke - Department of Engineering, Durham University, South Rd, Durham, DH1 3LE, UK. **E-mail**: michael.cooke@durham.ac.uk

Andrew. J. Gallant - Department of Engineering, Durham University, South Rd, Durham, DH1 3LE, UK. **E-mail**: a.j.gallant@durham.ac.uk
